# Effectiveness of Electroacupuncture for Simple Obesity: A Systematic Review and Meta-Analysis of Randomized Controlled Trials

**DOI:** 10.1155/2020/2367610

**Published:** 2020-06-28

**Authors:** Yanling Gao, Yi Wang, Jing Zhou, Zhihai Hu, Yin Shi

**Affiliations:** ^1^Graduate School, Shanghai University of Traditional Chinese Medicine, Shanghai 201203, China; ^2^Department of Acupuncture and Moxibustion, Shanghai TCM-Integrated Hospital, Shanghai University of TCM, Shanghai 200082, China; ^3^Key Laboratory of Acupuncture and Immunological Effects, Shanghai University of Traditional Chinese Medicine, Shanghai 201203, China; ^4^Outpatient Department, Shanghai Institute of Acupuncture-Moxibustion and Meridian, Shanghai 200030, China

## Abstract

**Objective:**

To evaluate the effectiveness of electroacupuncture in the treatment of simple obesity.

**Methods:**

Randomized clinical trials concerning electroacupuncture as a treatment of simple obesity published prior to October 31, 2019, were searched in the following Chinese and English databases: Chinese National Knowledge Infrastructure (CNKI), WanFang Database, China Science and Technology Journal Database (VIP), Chinese Biomedical Literature Database (CBM), PubMed, Cochrane Library, Web of Science, and Scopus. After data collection and quality evaluation, meta-analysis was performed using RevMan 5.3 software and Stata 15.0 software.

**Results:**

A total of 13 studies involving 937 patients with simple obesity were included in the meta-analysis. Results revealed that the total effective rate (RR = 1.29, 95% CI [1.13, 1.48]; *P*=0.0002), BMI (MD = −1.82, 95% CI [−2.21, −1.43]; *P* < 0.000), waist circumference (MD = −2.39, 95% CI [−3.95, −0.84]; *P*=0.003), hip circumference (MD = 0.31, 95% CI [−2.37, 2.99]; *P*=0.82), waist-hip ratio (MD = −0.05, 95% CI [−0.07, −0.03]; *P* < 0.00), and body fat rate (MD = −1.56, 95% CI [−2.35, −0.78]; *P*=0.0001) in the electroacupuncture group were superior to those in the control group. Analysis of acupoint clustering and correlation using SPSS 24.0 and Clementine 12.0 revealed the highest statistical support for acupoint groups CV12-CV4 and CV12-ST25-CV4, while ST36-CV12-ST25, SP6, and ST40-ST24-SP15-ST37-CV4 were found to be validly clustered acupoints.

**Conclusion:**

For treating simple obesity, electroacupuncture is superior to other interventions such as acupuncture, acupoint catgut embedding therapy, and simple lifestyle modification for improvement in body fat rate, waist circumference, and waist-hip ratio, although not hip circumference. Acupoint analysis revealed that ST25, CV12, CV4, SP6, and ST36 can form the basis for electroacupuncture therapy for the treatment of simple obesity.

## 1. Introduction

Recent decades have seen soaring obesity worldwide, in no small part due to lifestyle changes. According to the WHO, 13% of the world's adult population (11% of men and 15% of women) were obese in 2016, and worldwide obesity has nearly tripled since 1975 [1, 2]. Simple obesity refers to the absence of obvious neurological and endocrine etiologies and is primarily due to either excessive caloric intake or suboptimal energy consumption. This results in excess accumulation of body fat and, in turn, abnormal body weight. Although the etiologies of obesity remain undefined, diet, environment, poor antioxidant function, endocrine disorders, and heredity likely play major roles in its pathogenesis. In addition, excess weight and obesity have been reported to harm human health. Studies have reported that chronic disorders of metabolism are associated with an increased risk of cardiovascular disease, diabetes, hypertension, and kidney disease [3–5]. Obesity has even been implicated to be a cause of chronic migraines [6]. Furthermore, obesity has been found to affect the incidence and prognosis of various carcinomas [7–9]. Current treatments of obesity mainly include behavioral therapy, drugs, and surgery, with the latter two modalities especially possessing a large number of side effects. As one case study reported, use of certain weight-loss pharmacotherapies may affect control of HIV viral load [10]. Since obesity greatly impacts people's life and work habits and currently has limited treatment options available, researching safe and effective interventions with low rates of adverse reactions is of great significance.

Acupuncture, a form of traditional Chinese medicine (TCM), is widely accepted as an effective treatment for simple obesity due to its lack of significant side effects and relatively good tolerability. Electroacupuncture, a form of acupuncture where a small electric current is passed between pairs of acupuncture needles, is a common variant of this therapeutic modality. Studies have shown that electroacupuncture combined with treadmill exercise significantly increases expression of PGC-1*α*, restores healthier levels of fatty acid oxidation, and improves skeletal muscle mitochondrial function, thus contributing to weight loss [11]. Animal studies have suggested that electroacupuncture can reduce weight by decreasing the expression of IL-6 [12], changing the expression of irisin [13], and inhibiting food intake when electroacupuncture treatment at acupoint ST36 upregulated anorexigenic proopiomelanocortin production in the solitary and hypoglossal nuclei [14]. Although the aforementioned studies provided noteworthy evidence regarding the treatment of obesity by electroacupuncture, we searched for randomized clinical trials (RCT) concerning such a treatment regimen in eight databases and performed a systematic review as well as meta-analysis in order to report a more advanced objective validation of available data.

## 2. Methods

### 2.1. Study Selection

The included studies satisfied the following criteria: (1) clinical controlled trial (CCT) and RCT studies with at least 10 cases of simple obesity treated with electroacupuncture; (2) studies including patients diagnosed with simple obesity; (3) studies in which treatment group patients underwent electroacupuncture at any acupoints, were treated with any needling technique, and were treated for any duration, while control group patients were treated with any other regimen; (4) studies with full text available.

Exclusion criteria were as follows: (1) conference papers, reviews, and studies with control groups treated by electroacupuncture and those with more than two study groups; (2) studies with obvious errors or major shortcomings; (3) studies with unavailable full text.

### 2.2. Search Strategy

Eight databases, including China National Knowledge Infrastructure (CNKI), WanFang Database, Chinese Science and Technology Periodical Database (VIP), Chinese Biomedical Literature Database (CBM), PubMed, Cochrane Library, Web of Science, and Scopus, were searched from inception to October 31, 2019. Search terms were electroacupuncture, electroacupuncture therapy, simple obesity, obesity, randomized, and randomized clinical trials. An example search of PubMed is shown in Table 1.

### 2.3. Data Extraction

Author(s), publication year, sample sizes, interventions, randomization methods, blinding methods, baselines, diagnostic criteria, evaluation standards of therapeutic effects, inclusion and exclusion criteria, follow-up periods, dropout or withdrawal rates, adverse events, and safety indices were extracted from included literature by two individuals.

### 2.4. Quality Assessment

Bias risks of included studies were assessed according to the Cochrane Handbook version 5.1.0. Assessment included six aspects: (1) selection bias, (2) performance bias, (3) detection bias, (4) attrition bias, (5) selective reporting, and (6) other biases. Each aspect was evaluated and classified into three groups: (1) low risk (bias did not significantly affect results), (2) high risk (bias significantly impacted result credibility), and (3) unclear risk (the possibility of bias affecting results was suspected). Study quality was assessed by two evaluators; any disagreement was discussed with a third reviewer.

### 2.5. Statistical Analyses

RevMan 5.3 software and Stata 15.0 software were used for data analysis and publication bias assessment by detailing forest and funnel plots. The weighed mean difference (MD) for measured data and relative risk (RR) for enumerated data were used to assess study heterogeneity. If there was low heterogeneity between trials (*P* > 0.10; *I*^2^<50%), a fixed effects (FE) model was used; otherwise, a randomized effects (RE) model was applied. The RR with a 95% confidence interval (CI) was calculated; *P* values less than 0.05 were considered to be statistically significant.

## 3. Results

### 3.1. General Literature Description

**Figure 1:**
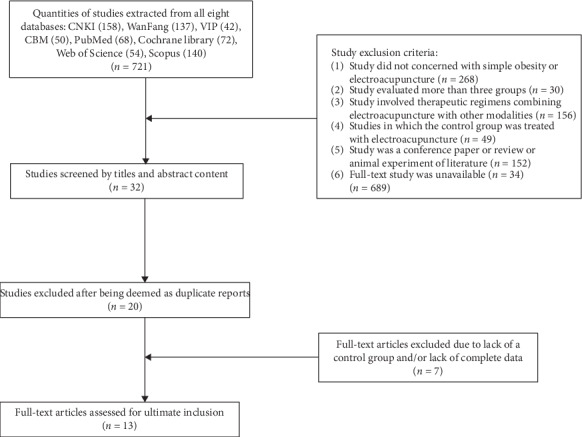
Flow chart of study extraction and selection processes.

Our search strategy resulted in a total initial extraction of 721 papers from all of the aforementioned eight databases, of which 387 were in Chinese (158 from CNKI; 137 from the WanFang Database; 42 from VIP, and 50 from CBM) and 334 were in English (68 from PubMed; 72 from the Cochrane Library; 54 from Web of Science, and 140 from Scopus). After ruling out duplication and study failure to meet our inclusion criteria, 13 papers, of which 13 were in Chinese ultimately included in our analysis (Figure 1).

### 3.2. Study Characteristics

**Figure 2:**
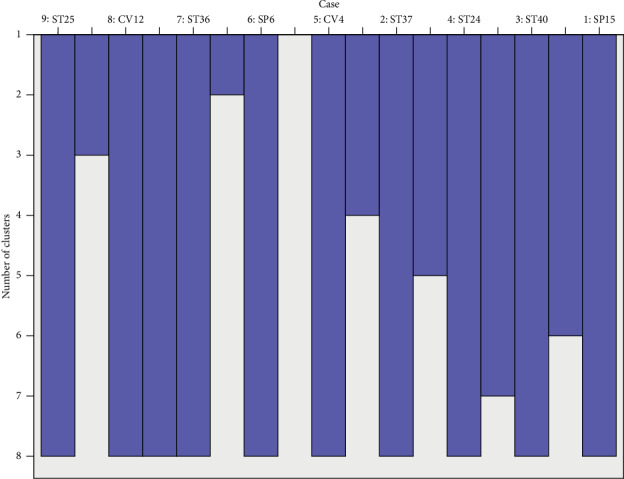
Icicle plot of main acupoints selected for electroacupuncture treatment of simple obesity.

**Figure 3:**
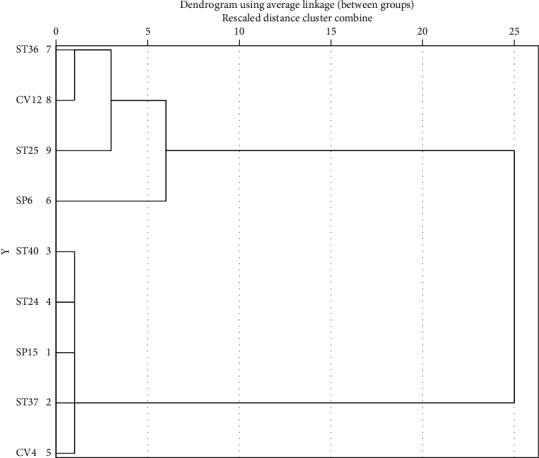
Dendrogram of main acupoints selected for electroacupuncture treatment of simple obesity.

Table 2 details characteristics of included studies. Altogether, the 13 papers we ultimately analyzed studied 937 participants, 470 of which were in an electroacupuncture group and 467 of which were in a control group. One paper compared the efficacy of electroacupuncture with that of acupoint catgut embedding [15], five with basic treatment [17, 19, 20, 23, 24], six with regular acupuncture [16, 18, 21, 22, 26], and one with sham electroacupuncture [25]. In all of these studies, 33 acupoints were used to treat simple obesity: Zhongwan (CV12), Qihai (CV6), Tianshu (ST25), Liangmen (ST21), Daimai (GB26), Daheng (SP15), Quchi (LI11), Zusanli (ST36), Xiajuxu (ST39), Shangjuxu (ST37), Fenglong (ST40), Shuifen (CV9), Guanyuan (CV4), Sanyinjiao (SP6), Shangwan (CV13), Xiawan (CV10), Zhigou (TE6), Fuai (SP16), Fujie (SP14), Huaroumen (ST24), Wailing (ST26), Shuidao (ST28), Zhangmen (LR13), Yinlingquan (SP9), Binao (LI4), Liangqiu (ST34), Gongsun (SP4), Pishu (BL20), Weishu (BL21), Daju (ST27), Neiting (ST44), Futu (ST32), and Yinjiao (CV7). The most frequently used 9 acupoints were ST 25 (12 times), ST36 (10 times), CV12 (9 times), SP6 (7 times), CV4 (5 times), SP15 (5 times), ST24 (5 times), ST37 (5 times), and ST40 (5 times). Association among main acupoints was analyzed using Clementine 12.0 software (Table 3). Support was defined as the ratio of literature mentioning the antecedent acupoints to that of all included papers. The first line of Table 3, for example, states that 40% of the 13 papers mentioned CV4 as a treatment acupoint. Confidence was defined as the accuracy and credibility of any given association; there thus was a 100% probability of papers mentioning CV12 as a treatment acupoint also mentioning CV4 as one. Greater confidence and support values revealed stronger association among acupoints. As seen in Table 3, acupoint groups with the highest support of 40% were CV12-CV4 and CV12-ST25-CV4. SPSS 21.0 software was used to analyze the clustering of acupoints selected to treat simple obesity by electroacupuncture at least five times. The icicle plot of Figure 2 details that there were three effective clusters. They were ST36-CV12-ST25, SP6, and ST40-ST24-SP15-ST37-CV4. The dendrogram of Figure 3 details the grading of the most commonly used acupoints into two groups: I, ST36-CV12-ST25, and II, ST40-ST24-SP15-ST37-CV4.

### 3.3. Quality of Included Studies

**Figure 4:**
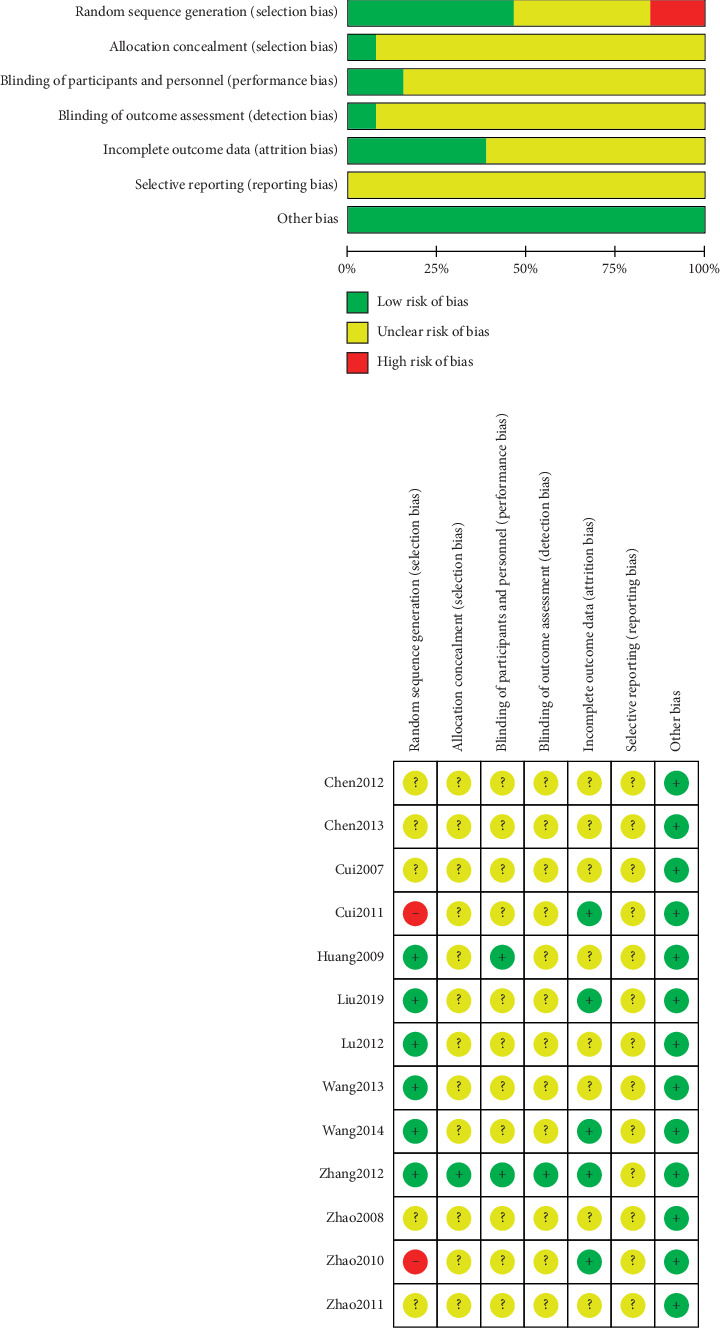
Risk of bias among included studies: determinations of review authors regarding risk of each bias item for each included study. “+,” low risk; “?,” unclear risk; “−,” high risk.

All 13 studies ultimately included in our analysis were of RCT and CCT design. Among them, six studies [15–17, 22, 25, 26] were determined to be at low risk for bias with use of a random number table; one study [23] with an odd/even random number and another [21] with the sequence of seeing doctors were determined to be at high risk for bias. Five studies [18–21, 24] that did not describe randomization methods were determined to be at unclear risk for bias. Only one study [25] describing allocation concealment was determined to be at low risk for bias while the remaining 12 not describing allocation concealment were considered to be at unclear risk. Two studies [22, 25] that reported blinding of both participants and personnel were determined to be at low risk for bias; the remaining studies not mentioning blinding methods were considered to be at unclear risk. Only one study [25] reported blinding of personnel to outcomes and was determined to be at low risk for bias; the remaining studies not mentioning such measures were considered to be at unclear risk. Three articles [21, 23, 25] that described dropout and loss to follow-up rates, as well as causes for these phenomena, and two [15, 17] not reporting such conditions were determined to be at low risk for bias. The remaining eight studies [16, 18–22, 24, 26] that did not describe outcome data in detail were considered to be at unclear risk. Although all fourteen studies described results, as primary statistical materials were not available, all 13 trials were determined to be at unclear risk for bias. The baseline between electroacupuncture and control groups in all trials was considered to be at low risk (Figure 4).

### 3.4. Outcome Meta-Analysis

#### 3.4.1. Overall Response Rate

**Figure 5:**
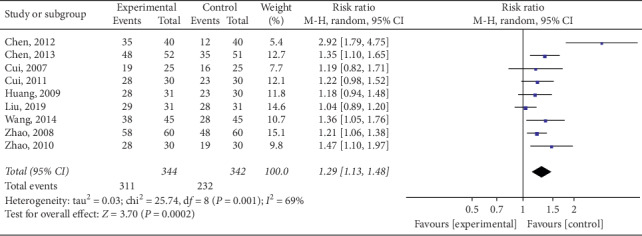
Meta-analysis of the overall response rate for the treatment of simple obesity with electroacupuncture.

**Figure 6:**
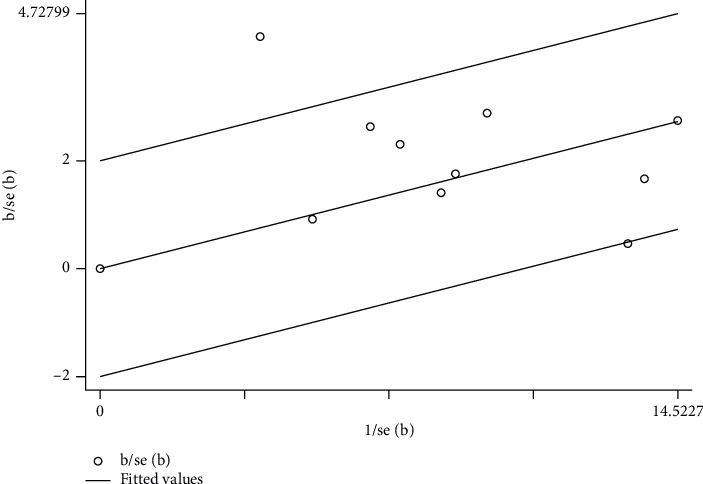
Meta-analysis on Galbraith of overall response rate in electroacupuncture treating simple obesity.

*(1) Tests for Heterogeneity*. Nine studies [15, 17, 18, 20–24] mentioned response rates in their outcomes; meta-analysis revealed a high heterogeneity (*P*=0.001; *I*^2^ = 69%). Thus, the random-effects model was used. As shown in Figure 5, electroacupuncture significantly differed from other interventions among control group patients, according to response rate (RR) analysis (pooled RR = 1.29, 95% CI [1.13, 1.48]; *P*=0.0002). The reconfirmation of heterogeneity on the basis of Galbraith may be caused by a study (Figure 6).

**Figure 7:**
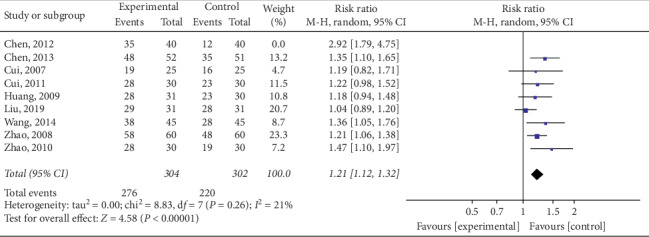
Meta-analysis concerning the sensitivity of the overall response rate for the treatment of simple obesity with electroacupuncture.

*(2) Sensitivity Analysis*. Sensitivity analysis was performed to identify the source of high heterogeneity. As shown in Figure 5, data from a prior study conducted by Chen et al. [20] revealed negligible overlapping of confidence intervals with data from another eight studies, suggesting data heterogeneity. As shown in Figure 6, one study's data was distributed outside the 95% confidence interval, likewise suggesting data heterogeneity. After excluding the study by Chen et al., heterogeneity decreased (*P*=0.026; *I*^2^ = 21%), as shown in Figure 7. In the detailed review of Chen et al.'s study in its entirety, we found that the data heterogeneity was primarily influenced by variation in studied parameters and differing intervention measures. Differences in studied parameters included varying subject inclusion and exclusion criteria or the differences caused by study sizes; disparities in intervention measures included data discrepancies caused by variations in patient compliance.

**Figure 8:**
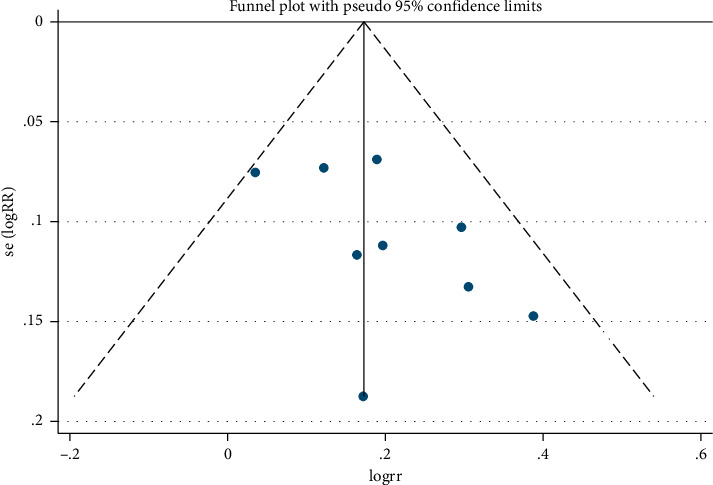
Meta-analysis on Funnel plot of overall response rate in electroacupuncture treating simple obesity.

*(3) Publication Bias*. Funnel plot (Figure 8) and Egger's test (Table 4) show that (*P*=0.107 > 0.05) there was no publication bias in the studies.

#### 3.4.2. Reduction of BMI

**Figure 9:**
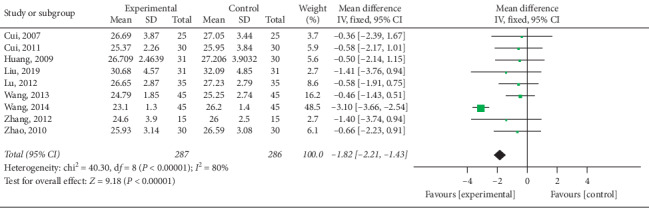
Meta-analysis concerning BMI reduction in the treatment of simple obesity with electroacupuncture.

**Figure 10:**
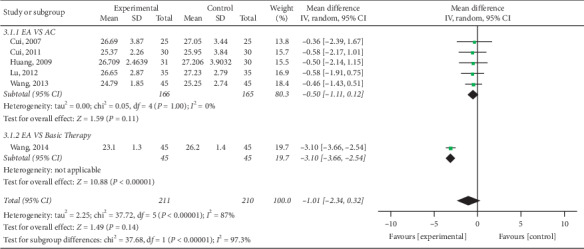
Comparison of BMI reduction via intervention subgroup analysis in the treatment of simple obesity with electroacupuncture.

**Figure 11:**
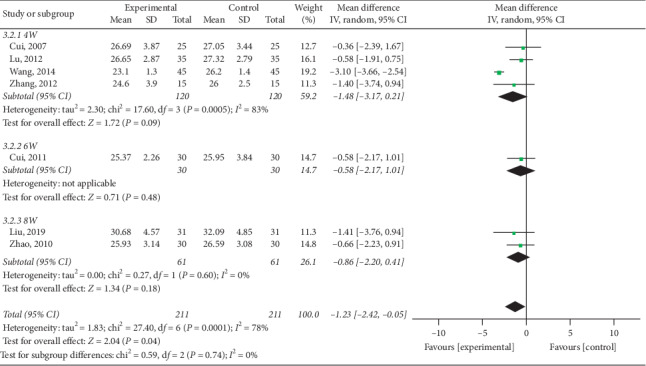
Comparison of BMI reduction via treatment course subgroup analysis in the treatment of simple obesity with electroacupuncture.

Nine trials [15–17, 21–23, 25, 26] mentioned BMI in their outcomes and meta-analysis revealed high heterogeneity among trials (*P* < 0.000; *I*^2^ = 80%). As illustrated in Figure 9, electroacupuncture was more effective than other interventions among control group patients in reducing BMI (pooled MD = −1.82, 95% CI [−2.21, −1.43]; *P* < 0.000). Due to high heterogeneity, subgroup analysis was conducted in terms of intervention (Figure 10) and treatment course (Figure 11). Heterogeneity was found to remain unaltered, although no source for it was identified.

#### 3.4.3. Reduction of Waist and Hip Circumference

**Figure 12:**
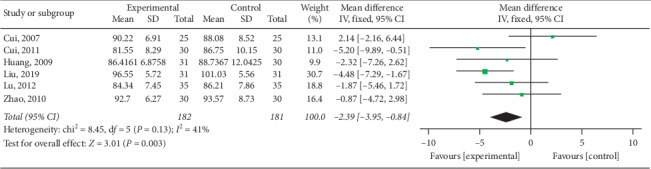
Meta-analysis of waist circumference reduction in the treatment of simple obesity with electroacupuncture.

**Figure 13:**
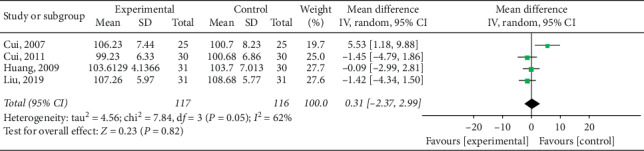
Meta-analysis of hip circumference reduction in the treatment of simple obesity with electroacupuncture.

Waist circumference (WC) was mentioned in the outcomes of six studies [15, 16, 21–23] and low heterogeneity was revealed in subsequent meta-analysis (*P*=0.13; *I*^2^ = 41%). Results revealed that electroacupuncture was superior to other interventions among control group patients in the reduction of waist circumference, as shown in Figure 12 (pooled MD = −2.39, 95% CI [−3.95, −0.84]; *P*=0.003). Four studies [15, 21, 22] reported hip circumference (HC) in their results; subsequent meta-analysis revealed high heterogeneity among trials (*P*=0.05; *I*^2^ = 62%) and no significant differences between electroacupuncture and control groups in the reduction of HC (pooled MD = 0.31, 95% CI [−2.37, 2.99]; *P*=0.82), as shown in Figure 13.

#### 3.4.4. Reduction of Waist-Hip Ratio

**Figure 14:**
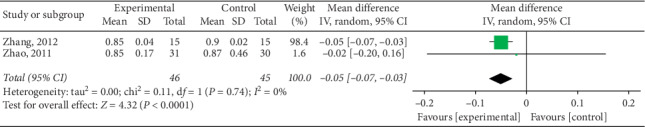
Meta-analysis of waist-hip ratio reduction in the treatment of simple obesity with electroacupuncture.

Only two trials [19, 25] mentioned the waist-hip ratio (WHR) in their results; subsequent meta-analysis revealed low heterogeneity (*P*=0.74; *I*^2^ = 0%). As illustrated in Figure 14, WHR reduction was more effective among electroacupuncture group patients when compared to control subjects (pooled MD = −0.05, 95% CI [−0.07, −0.03]; *P* < 0.00).

#### 3.4.5. Improvement of TCM Syndrome Score

**Figure 15:**
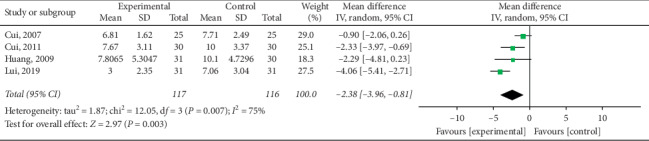
Meta-analysis of TCM syndrome score improvement in the treatment of simple obesity with electroacupuncture.

Statistical analysis of four articles [15, 21, 22] reporting TCM syndrome scores revealed high heterogeneity (*P*=0.007; *I*^2^ = 75%) and a significant difference among electroacupuncture and control group patients in the improvement of TCM syndrome scores (pooled MD = −2.38, 95% CI [−3.96, −0.81]; *P*=0.003). As illustrated in Figure 15, electroacupuncture was more effective than other interventions in control group patients.

#### 3.4.6. Body Fat Rate Reduction (BF%)

**Figure 16:**
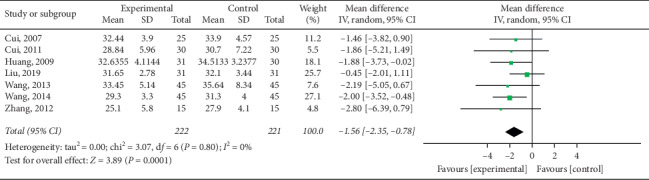
Meta-analysis of body fat rate reduction in the treatment of simple obesity with electroacupuncture.

Seven studies [15, 17, 21, 22, 25, 26] reported body fat rate in their outcomes. Statistical analysis revealed high heterogeneity (*P*=0.80; *I*^2^ = 0%). As shown in Figure 16, electroacupuncture group patients were found to have had greater obesity index reduction when compared to control group subjects (pooled MD = −1.56, 95% CI [−2.35, −0.78]; *P*=0.0001).

## 4. Discussion

Obesity, one of the most serious conditions harming public health over the world, is the main etiology of type 2 diabetes mellitus and cardiovascular disease [27]. Unfortunately, to date, no safe and effective pharmacotherapy has been found to treat obesity [28]. Therefore, study of novel and alternative therapeutic modalities is of great importance. Acupuncture, a form of TCM, has been previously reported effective in treating obesity [29–32]. Furthermore, recent years have witnessed an increasing number of clinical studies concerning the treatment of obesity using electroacupuncture [33–35]. Electroacupuncture was found to promote fat metabolism via sympathetic nervous system activation, thus decreasing appetite [36]. To confirm the effectiveness of this therapy as a treatment of simple obesity and help establish a foundation for clinical guideline formation, we conducted this systematic review and meta-analysis evaluating published RCT and CCT data available in major scientific article databases.

Thirteen trials were included in analysis after rigorous screening. Meta-analysis was employed to analyze response rates reported in nine trials, BMI in nine, WC in six, HC in four trials, WHR in two, TCM syndrome scores in four, and body fat rate in seven. The results revealed electroacupuncture to be more effective than other therapies in improving the TCM syndrome score (*P*=0.003) and overall response rate (*P*=0.0002) as well as reducing BMI (*P* < 0.000), WC (*P*=0.003), WHR (*P* < 0.00), and the body fat rate (*P*=0.0001). However, no superiority of electroacupuncture in the reduction of HC was noted (*P*=0.82). Overall response rate analysis revealed high heterogeneity (*P*=0.001; *I*^2^ = 69%). Detailed, individual analysis of nine trials revealed that high heterogeneity was likely due to a study by Chen et al. [20]; heterogeneity decreased (*P*=0.26; *I*^2^ = 21%) after exclusion of this study. In addition, there was high heterogeneity (*P* < 0.000; *I*^2^ = 89%) among trials in regard to BMI reduction. To identify the cause of this phenomenon, subgroup analysis was performed, revealing no source for the heterogeneity and that it remained unchanged in terms of intervention (*P* < 0.000; *I*^2^ = 87%) and treatment course (*P*=0.0001; *I*^2^ = 78%). Overall analysis of 13 studies revealed electroacupuncture to be more effective than acupuncture, acupoint catgut embedding, and basic treatment in the therapeutic management of simple obesity.

We also studied the association among main acupoints selected for treatment. Acupoint groups with the highest statistical support were found to be CV12-CV4 and CV12-ST25-CV4, followed by CV6-SP15, CV6-CV12-SP15, ST26-ST24, ST26-ST25-ST24, ST24-ST26, ST24-ST25-ST26, SP15-CV6, SP15-CV12-CV6, ST26-ST25-SP15, ST26-CV12-ST25-SP15, ST24-ST25-SP15, ST24-CV12-ST25-SP15, CV6-ST25-SP15, and CV6-CV12-ST25-SP15. Clustering analysis revealed three effective clusters; namely, ST36-CV12-ST25, SP6, and ST40-ST24-SP15-ST37-CV4. Our findings also suggested that ST25-CV12-CV4-SP6-ST36 serves as the primary acupoint group for effective electroacupuncture treatment of obesity, with ST24, ST26, ST37, SP15, and ST40 serving as supplementary acupoints capable of treating different TCM syndromes associated with simple obesity.

This study did not lack shortcomings with the potential to limit our above conclusions. As all included trials were written in Chinese and no articles in other languages were initially searched for, the possibility of publication bias must be considered. All RCT data containing negative results were excluded; this could have potentially caused overestimation of response rates and given rise to result bias. Only one included study employed allocation concealment and two employed blinding, implying that the majority of studies we evaluated were low in quality. Inclusion of low-quality data compiled using nonstandardized diagnostic and evaluation criteria may have likewise led to publication bias. When high heterogeneity was detected, sensitivity and subgroup analyses were successful in only partially revealing sources of heterogeneity.

Rigorous study design including characteristics such as allocation concealment, adequate blinding, employment of randomization, and consideration of adverse effects, as well as detailed discussion of negative results, should be encouraged in future electroacupuncture research. More detailed information regarding electroacupuncture therapy, such as needling sites, insertion depths, needle types, and acupoints selected are likewise of great importance in the future establishment of clinical electroacupuncture therapy guidelines.

## 5. Conclusion

In the treatment of simple obesity, electroacupuncture is superior to acupuncture, acupoint catgut embedding, and basic treatment in improving the body fat rate, BMI, WC, WHR, and TCM syndrome scores, but not HC. Acupoint analysis revealed ST25, CV12, CV4, SP6, and ST36 to be most effective in the treatment of obesity with electroacupuncture.

## Figures and Tables

**Table 1:** PubMed search strategy.

Search	Query	Item found
#12	Search((((electroacupuncture) OR “Electroacupuncture” [MESH]))	68
	AND (((obesity) OR simple obesity) OR “obesity” [Mesh]))AND(((	
	random^*∗*^) OR “Randomized Controlled Trials as Topic” [MESH]) OR	
	randomized controlled trial [Publication Type])	
#11	Search((random^*∗*^) OR “Randomized Controlled Trials as Topic”	1312396
	[MESH]) OR randomized controlled trial [Publication Type]	
#10	Search randomized controlled trial [Publication Type]	493918
#9	Search“Randomized Controlled Trials as Topic” [MESH]	130784
#8	Search random^*∗*^	1311522
#7	Search (electroacupuncture) OR “electroacupuncture” [MESH]	5153
#6	Search “electroacupuncture” [Mesh]	3756
#5	Search electroacupuncture	5153
#4	Search((obesity) OR simple obesity) OR “obesity” [Mesh]	318122
#3	Search simple obesity	5280
#2	Search “obesity” [Mesh]	203151
#1	Search obesity	318122

**Table 2:** Characteristics of included studies.

Study ID	N		Interventions		Treatment (W)	Main acupoints
	E	C	E	C		
Liu [15]	31	31	EA	ACE	8	CV12, CV6, ST25, ST21, GB26, SP15, LI11, ST36, ST39, ST37, ST40
Lu et al. [16]	35	35	EA	CA	4	CV13, CV12, CV10, CV9, CV6, CV4, SP16, SP14, SP15, ST21, ST24, ST25, ST26, ST28, LR13, ST36, SP9, SP6, LI11, LI4
Wang and Xiao [17]	45	45	EA	BT	4	CV12, CV4, SP15, CV10, CV6, ST25, ST24, ST26
Zhao et al. [18]	60	60	EA	CA	6	ST34, SP4, ST37, ST25
Zhao et al. [19]	31	30	EA	BT	7	CV12, ST25, CV4, ST36, ST40, SP9, SP6, BL20, BL21
Chen et al. [20]	40	40	EA	BT	4	ST25, TE6, SP6, ST36, ST40, SP9, ST27
Cui [21]	25	25	EA	CA	4	CV9, CV7, ST26, ST25, ST24
Huang [22]	31	30	EA	CA	5	CV4, CV12, ST25, SP14, GB26, LI11, ST36, SP6, ST40, ST44, TE6, ST37, ST39
Zhao and Shi [23]	30	30	EA	BT	8	ST25, ST24, ST26 , ST32, ST36, ST37, ST39, ST44, SP6, SP15, SP14, CV12, CV10, CV4
Chen [24]	52	51	EA	BT	4	CV12, CV6, ST24, SP15, TE6, ST36, ST34, SP6
Zhang et al. [25]	15	15	EA	SEA	4	ST25, SP6, CV12, ST36
Wang et al. [26]	45	45	EA	CA	12	ST44, ST37, ST39, ST40, ST25, ST36, SP9, LI11
Cui [21]	30	30	EA	CA	6	CV12, ST25, GB26, ST28, ST36

Notes: E, experimental group; C, control group; W, week; EA, electroacupuncture; ACE, acupoint catgut embedding; BT, basic treatment; CA, acupuncture.

**Table 3:** Association among main acupoints selected for the treatment of simple obesity by electroacupuncture.

Consequent	Antecedent	Support (%)	Confidence (%)
CV12	CV4	40	100
CV12	ST25 and CV4	40	100
CV6	SP15	33.33	80
CV6	CV12 and SP15	33.33	80
ST26	ST24	26.67	100
ST26	ST25 and ST24	26.67	100
ST24	ST26	26.67	100
ST24	ST25 and ST26	26.67	100
SP15	CV6	26.67	100
SP15	CV12 and CV6	26.67	100
ST26	ST25 and SP15	26.67	75
ST26	CV12 and ST25 and SP15	26.67	75
ST24	ST25 and SP15	26.67	75
ST24	CV12 and ST25 and SP15	26.67	75
CV6	ST25 and SP15	26.67	75
CV6	CV12 and ST25 and SP15	26.67	75
ST26	CV4 and ST24	20	100
ST26	SP15 and ST24	20	100
ST26	SP15 and CV4	20	100
ST26	ST25 and CV4 and ST24	20	100
ST26	ST25 and SP15 and ST24	20	100
ST26	ST25 and SP15 and CV4	20	100
ST26	CV12 and ST24	20	100
ST26	CV12 and CV4 and ST24	20	100
ST26	CV12 and SP15 and ST24	20	100
ST26	CV12 and SP15 and CV4	20	100
ST26	CV12 and ST25 and ST24	20	100
ST24	CV4 and ST26	20	100
ST24	SP15 and ST26	20	100
ST24	SP15 and CV4	20	100
ST24	ST25 and CV4 and ST26	20	100
ST24	ST25 and SP15 and ST26	20	100
ST24	ST25 and SP15 and CV4	20	100
ST24	CV12 and ST26	20	100
ST24	CV12 and CV4 and ST26	20	100
ST24	CV12 and SP15 and ST26	20	100
ST24	CV12 and SP15 and CV4	20	100
ST24	CV12 and ST25 and ST26	20	100
CV4	SP15 and ST26	20	100
CV4	SP15 and ST24	20	100
CV4	ST25 and SP15 and ST26	20	100
CV4	ST25 and SP15 and ST24	20	100
CV4	CV12 and ST26	20	100
CV4	CV12 and ST24	20	100
CV4	CV12 and SP15 and ST26	20	100
CV4	CV12 and SP15 and ST24	20	100
CV4	CV12 and ST25 and ST26	20	100
CV4	CV12 and ST25 and ST24	20	100

**Table 4:** Egger's test.

Std_Eff	Coef.	Std. err.	t	*P* > |*t*|	[95% conf. interval]
Slope	0.000826	0.097612	0.01	0.993	−0.2299902 0.2316429
Bias	1.856624	1.004787	1.85	0.107	−0.5193202 4.232569
